# Management of large and surrounding tissue infiltrative anal fistula cancer: Two case reports

**DOI:** 10.1016/j.ijscr.2020.11.107

**Published:** 2020-11-21

**Authors:** Sang Hyun An, Ik Yong Kim

**Affiliations:** Department of Surgery, Yonsei University Wonju College of Medicine, Wonju, South Korea

**Keywords:** Anal fistula, Adenocarcinoma, Abdominoperineal Resection

## Abstract

**Introduction:**

Adenocarcinoma originating from chronic perianal fistulas is a rare disease. Due to the lack of evidence of this malignancy, no definitive diagnostic and treatment guidelines have been established. We present two cases of anal fistula cancer and discuss the diagnostic and treatment strategies.

**Presentation of case:**

A 79-year-old man was referred for a perianal mass. The patient had a history of chronic perianal fistula and was diagnosed with adenocarcinoma originating from a long-standing perianal fistula. The patient underwent laparoscopic abdominoperineal resection (APR) without any adjuvant therapy. The second case was a 42-year old man, who was referred for a prolapsed anal mass. The patient had recurrent perianal fistula over several years. He was diagnosed with mucinous adenocarcinoma originating from a long-standing perianal fistula, but he initially refused surgical treatment. Two years later, the patient revisited with a huge overgrown tumor and underwent laparoscopic APR with wide perineal skin excision, wound debridement and coverage with a bilateral V-Y advancement flap via a one-step procedure.

**Discussion:**

High degree of clinical suspicion is crucial to diagnose this rare disease that can easily be missed at an early stage. Depending on the disease stage, surgical treatment, chemotherapy, and radiotherapy can be considered.

**Conclusion:**

Histopathological evaluation should be performed on recurrent and incurable anal fistulas over a long period of time. Intensive surgical treatment with or without neoadjuvant and adjuvant therapy should be considered in advanced cases.

## Highlights

• We reported two cases of adenocarcinoma originating from anal fistula.
• High degree of clinical suspicion is crucial to diagnose anal fistula cancer.
• Histopathological evaluation should be performed on recurrent, incurable anal fistulas.
• Intensive surgical treatment with neoadjuvant or adjuvant therapy should be considered in advanced cases.

## 1 Introduction

Anal cancer is a rare malignancy that accounts for approximately 3% of all gastrointestinal malignancies [1]. It is even rarer to find adenocarcinoma originating from long-standing perianal fistulas. In 1934, Rosser C. was the first to report the association of perianal carcinoma with a long-standing anal fistula in seven cases [2]. Because of its rarity and lack of existing literature, the pathogenesis, biological behavior, and treatment of the disease have not been established yet. The diagnosis is difficult because this type of malignancy can be misdiagnosed as a common benign disease such as perianal abscess and fistula. Complete resection, such as abdominoperineal resection (APR), has been considered as the standard surgical method, however, it is difficult due to its large tumor size and surrounding tissue infiltration. In addition, controversies still exist regarding the use of neoadjuvant or adjuvant chemo-radiotherapy followed by surgery [[3], [4], [5], [6]]. Here, we describe two cases of adenocarcinoma arising from perianal fistula, which were successfully resected by laparoscopic APR in one case and laparoscopic APR with bilateral V-Y advancement flap in the other. This case report has been reported in line with the SCARE 2018 criteria [7].

## 2 Presentation of case

### 2.1 Case 1

**Fig. 1:**
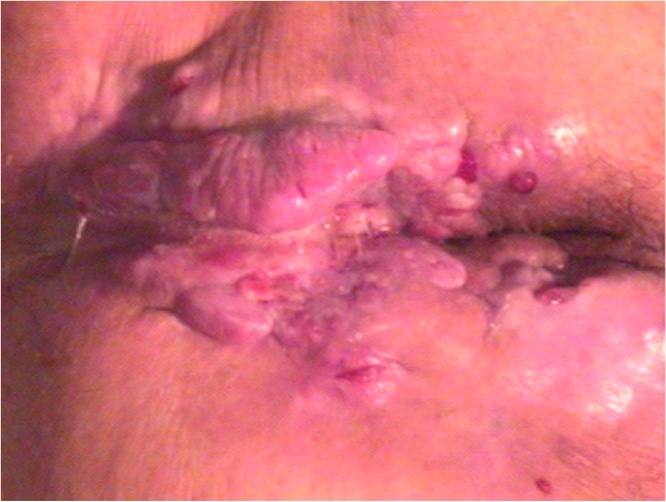
Perianal mass. Large, irregular perianal ulcerated mass with multiple fistula tracts.

**Fig. 2:**
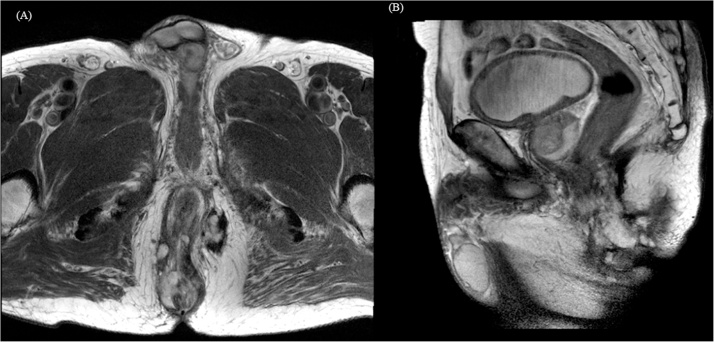
Magnetic resonance image (MRI) findings. MRI showed perianal fistula and associated perianal multifocal abscess cavity. There was no evidence of lymphadenopathy in perirectal space, both internal iliac vessel area. (a) T2 weighted Magnetic resonance imaging. Axial view. (b) T2 weighted Magnetic resonance imaging. Sagittal view.

A 79-year-old male presented with symptoms of anal pain, change in bowel habits, and perianal mass with anal discharge for one year, and was referred to our hospital. He had a history of undergoing necrotherapy for recurrent internal hemorrhoids 40 years ago. Physical examination revealed a large irregular perianal ulcerated mass with multiple fistula tracts (Fig. 1). During colonoscopy, multiple ulcerated lesions with discharge were observed around the anus. Magnetic resonance imaging (MRI) showed perianal multifocal abscess, however, there were no findings indicating malignant characteristics or lymphadenopathy (Fig. 2). Excisional biopsy of the mass was performed via the transanal approach and the pathologic findings were suggestive of adenocarcinoma. The initial serum carcinoembryonic antigen (CEA) level was 59.81 ng/mL. Patient underwent laparoscopic APR, and pathologic findings of the operated mass were reported as 7 cm × 4 cm × 3 cm well-differentiated adenocarcinoma arising in an anal fistula with invasion of perianal soft tissue and skin without lymph node metastasis (pT3 N0, LN = 0/18). The postoperative course was uneventful, and he was discharged without any postoperative complications. He had a favorable outcome without any evidence of recurrence or distant metastasis, at 3 years of follow-up.

### 2.2 Case 2

**Fig. 3:**
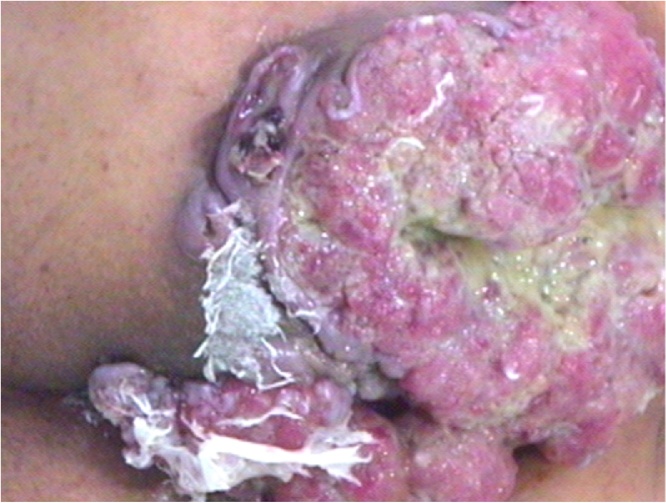
Perianal mass. Two years later from initial visiting. Huge multinodular mass with mucinous material in anal canal with extension to the subcutaneous tissue and overlying skin.

**Fig. 4:**
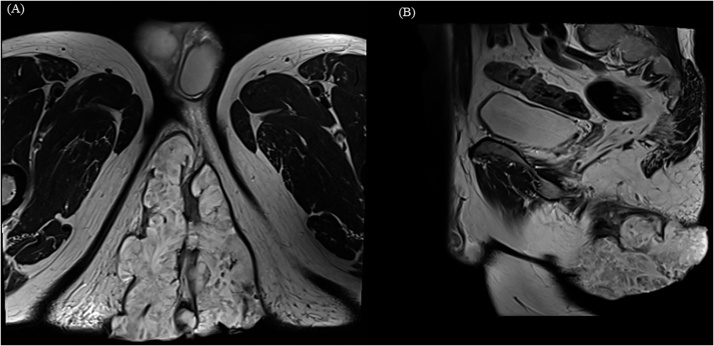
MRI findings. MRI showed 12 × 11 cm sized huge mass with extension to external anal sphincter and levator ani muscle. There was no evidence of lymphadenopathy in perirectal space and both pelvic wall. (a) T2 weighted Magnetic resonance imaging. Axial view. (b) T2 weighted Magnetic resonance imaging. Sagittal view.

**Fig. 5:**
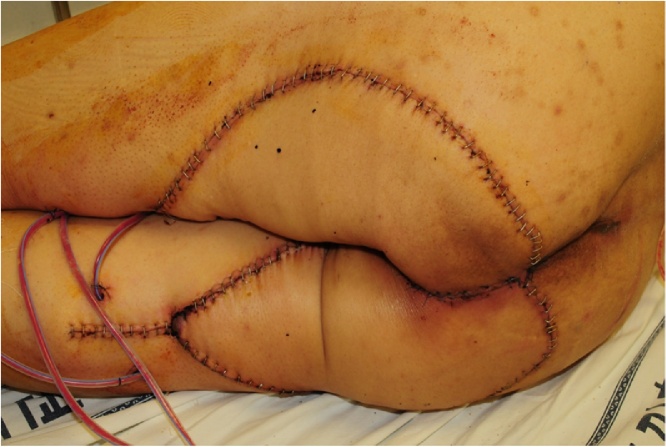
Postoperative wound. Bilateral V-Y advancement flap.

A 67-year-old man with prolapsed anal mass, who had a medical history of hypertension, diabetes mellitus, and endoscopic submucosal dissection (ESD) for early gastric cancer was referred to our hospital. He had suffered from recurrent fistula in the anus over several years and had been symptomatic with anal pain and discharge. Physical examination revealed external openings at 6–9 o'clock position around the anus. Before coming to our clinic, he underwent incision and drainage with biopsy at a local hospital, and pathology revealed adenocarcinoma with mucinous differentiation. We planned APR with the possibility of a malignancy of chronic fistula, but the patient refused surgery because of fear of excision of anal sphincter and permanent stoma. Two years later, the patient revisited our clinic with a rapidly growing perianal mass and aggravation of symptoms. The mass extended widely in the perineum and buttock areas (Fig. 3). MRI showed 11.8 cm × 10.1 cm sized mass in the perineal area, which penetrated external anal sphincter and levator-ani muscle without any evidence of lymph node involvement in the pelvic cavity (Fig. 4). Tumor markers were identified in the normal range. The patient underwent laparoscopic APR with wide perineal skin excision, wound debridement and coverage with bilateral V-Y advancement flap via a one-step procedure (Fig. 5). Pathologic findings were reported as 12 cm × 11 cm × 9 cm moderately differentiated mucinous adenocarcinoma arising from an anal fistula, which extended to the sphincter muscle and perianal skin without lymph node metastasis (pT3 N0, L/N = 0/23). He was discharged on the 22nd day after the index surgery without any complications. After surgery, he received chemotherapy using a regimen of intravenous 5-fluorouracil for 6 months as adjuvant therapy. The patient experienced a favorable outcome during 4 years of follow-up without any local recurrence or distant metastasis.

## 3 Discussion

Although anal fistula is a common benign disease in coloproctology, perianal adenocarcinoma associated with chronic fistula-in-ano is rare, and has been reported in approximately less than 200 cases in the literature [8]. Therefore, there has been no definitive consensus on the pathogenesis, diagnosis, and treatment of the disease. Although the exact pathogenesis of the disease is not well known, it is believed to be associated with chronic inflammation. Chronic inflammation induces tissue damage, and active cell proliferation occurs during the healing process. Repeated tissue damage and regeneration results in permanent genetic mutations, and activated inflammatory cells produce various chemokines and cytokines, which can influence tumor growth, migration, and differentiation [[9], [10], [11]].

Patients are usually diagnosed at an advanced stage because symptoms are similar to benign anal diseases and progress slowly. Endoscopic findings cannot usually provide definite diagnostic evidence as the tumor does not pierce the rectal mucosa. Rosser C. was the first to publish seven cases of anal fistula cancer in 1934; thereafter, several researchers have reported case series and established the following three diagnostic criteria for malignancy of anal fistula: (1) the fistula should usually antedate the carcinoma by at least 10 years, (2) the only tumor present in the rectum or anal canal should be secondary to direct extension from the carcinoma in the fistula, and (3) the internal opening of the fistula should be into the anal canal and not into the tumor itself [2,12,13]. The definite diagnosis of the disease can be established by histopathological examination through biopsy, and imaging studies including CT scan and MRI provide information about disease invasion into adjacent tissues. In cases of mucinous adenocarcinoma, pelvic MRI is a useful imaging modality because it can show a significant hyper-intense signal on T2-weighted images [14].

Surgical resection has been considered the standard treatment option. APR is the most preferred surgical method, and in advanced cases, wide excision of surrounding tissues including the overlying skin is necessary in order to secure a negative resection margin. In advanced cases, chemotherapy, radiotherapy, and chemoradiotherapy (CRT) can be considered as neoadjuvant or adjuvant therapy, however, its effectiveness remains controversial. According to a study of 14 patients with anal fistula malignancy conducted by Gaertner et al., neoadjuvant chemoradiotherapy or adjuvant chemotherapy was found to be an effective treatment option for these patients, as 7 of 14 patients who received neoadjuvant CRT in this study had a complete response [15]. Hongo et al. conducted a study with 11 patients, and they concluded that patients who underwent multimodality therapy including neoadjuvant CRT or radiotherapy had better relapse-free survival [5]. Santos et al. reported that preoperative CRT could cause tumor regression and downsizing, thus increasing the chances of complete radical resection in locally advanced disease [4].

According to previously published literature, metastases to lymph nodes may be present in advanced cases, and inguinal lymph nodes are the most frequent sites of metastasis, whereas distant metastasis is uncommon. Gaertner et al. reported that 6 of the 14 patients (43%) had nodal involvement, and suggested that nodal involvement was associated with decreased survival [15]. The prognosis is known to be poor when the tumor is larger than 5 cm, CEA is elevated, or any metastasis including lymph node involvement are present at the time of diagnosis [8,15,16]. However, there have been no established long-term survival and recurrence data of this disease due to the lack of large-scale studies and literature.

In our case series, both patients were found to meet the diagnostic criteria, and the diagnosis was confirmed by preoperative histological examination. One patient was identified as a case of mucinous adenocarcinoma, and the other was identified as a case of adenocarcinoma. No lymph node metastasis was observed in either of the patients. One patient received only APR, and the other received adjuvant chemotherapy after curative resection. Several studies have reported that mucinous carcinomas have a poorer prognosis than non-mucinous tumors in colorectal cancer [17,18]. However, to our knowledge, there have been no studies on the difference in disease progression between these two types of tumors originating from chronic perianal fistula, and their prognosis is unknown. Both the patients in our case series showed favorable progress without any local recurrence or distant metastasis.

## 4 Conclusion

To summarize, high degree of clinical suspicion is crucial to diagnose this rare disease that can easily be missed at early stage. Besides, it should be noted that fistulectomy for preventing purpose and the histopathological evaluation should be performed on recurrent and incurable anal fistulas over a long period of time. These tumors have a high potential for local recurrence, therefore, curative surgical treatment should be carried out, and multimodal approaches including neoadjuvant or adjuvant CRT should also be considered in advanced cases. Furthermore, large-scale studies should be performed to establish knowledge related to the treatment and prognosis of this disease.

## Declaration of Competing Interest

The authors report no declarations of interest.

## Funding

This paper did not receive any specific grant from funding agencies in the public, commercial, or not-for profit sectors.

Sang Hyun An: Nothing to declare.

Ik Yong Kim: Nothing to declare.

## Ethical approval

This is case report study and ethical approval not required.

## Consent

Written informed consent was obtained from the patients for publication of this case report and accompanying images. A copy of the written consent is available for review by the Editor-in-Chief of this journal on request.

## Registration of research studies

The following statement applies for all listed authors:

This case report does not require registration.

This is a case report.

## Guarantor

Ik Yong Kim, M.D., Ph.D.

## Provenance and peer review

Not commissioned, externally peer-reviewed.

## CRediT authorship contribution statement

**Sang Hyun An:** Writing - original draft, Writing - review & editing. **Ik Yong Kim:** Conceptualization, Data curation, Project administration, Supervision.
